# Analyzing collaboration dynamics in new drug R&D: A case study of two lipid-lowering drugs

**DOI:** 10.3389/fphar.2025.1516882

**Published:** 2025-05-30

**Authors:** Nan Zhang, Huaqing Guo, Mengchao Zhang, Chen Wang, Ruonan Tian, Yan Shi, Zhiguang Duan

**Affiliations:** ^1^ Department of Health Administration, School of Management, Shanxi Medical University, Taiyuan, China; ^2^ Department of Humanistic Medicine, School of Humanities and Social Sciences, Shanxi Medical University, Taiyuan, China

**Keywords:** new drug, research and development, academic chain, collaborative networks, research management

## Abstract

**Introduction:**

The advancements in biotechnology have ushered in a new age for drug development, characterized by increased collaborative efforts. Academic institutions, pharmaceutical firms, hospitals, foundations, and various other entities across different sectors are now joining forces more frequently to accelerate new drug innovation. However, there remains a limited understanding of how scientific and technological advancements are influencing these research collaborations.

**Methods:**

In this study, the development of two types of lipid-lowering drugs served as case studies. A detailed network analysis was performed at the levels of authors, institutions, and countries to quantify the evolutionary trends in research collaboration.

**Results:**

In the clinical research segment of the academic chain, papers resulting from collaborations tend to receive a higher citation count compared to other areas. However, there were notably fewer collaborative connections between authors transitioning from basic to developmental research and beyond. Collaboration models involving universities with enterprises, hospitals, or both are becoming more prevalent in biologics R&D. These models demonstrate effects of similarity and proximity. Additionally, there has been an increase in the involvement of developing countries in the research and development of new biologic drugs on a national and regional scale.

**Conclusion:**

New drug R&D research collaboration patterns evolve spontaneously with productivity updates. In the future, it is necessary to enhance the involvement of pharmaceutical companies in the basic research phase of new drug development, continuously strengthen the relationships across all segments of the academic chain, and thoroughly boost the efficiency of transforming new drug R&D into practical applications.

## 1 Introduction

New drug research and development encompasses basic research, drug development, preclinical studies, and clinical trials. This process is marked by high costs, lengthy timelines, and significant risks, yet it is crucial for enhancing disease treatment, extending patient lifespans, advancing scientific and technological progress, and boosting international competitiveness (Nicolaou, 2014; Janero, 2024). The 2023 White Paper on New Drugs Class I in China indicates that 1,051 new drugs have been approved, with biologics accounting for a proportion that is approximately equal to that of chemical drugs (49% vs 51%). With the approval of 30 new drugs for marketing, the number of biologics exceeded that of chemical drugs for the first time (16 vs 14), indicating that biologics have become a new trend in new drug development.

The initial step in new drug discovery often involves identifying novel drug targets, such as proteins and nucleic acids, which can be influenced by drugs. Academic institutions, as centers of research innovation, consistently pioneer the discovery of new drug targets and expand disease knowledge (Loregian and Palù, 2013; Everett, 2015). Upon identifying a new target, an extensive drug development program can commence, utilizing animal models and progressing to human clinical trials, typically spearheaded by the pharmaceutical industry. Large pharmaceutical companies bring to bear considerable expertise and technological platforms, extensive compound databases, substantial financial resources, and broad drug pipelines, enhancing the efficiency of R&D and spreading risks (Tralau-Stewart et al., 2009; Tian et al., 2021). Among the “China New” Top 30 targets in 2023, 84.5% of the drugs are still in clinical phase I and clinical phase II development in global pharmaceutical companies. These targets are undergoing in-depth clinical validation and efficacy evaluation. A mere 1.3% of these have been submitted for marketing authorization.

Insufficient validation of early-stage drug targets can escalate the risk of clinical trial failures and diminish drug approval rates, making thorough target validation a vital component of drug discovery (Dowden and Munro, 2019). Strategies used by pharmaceutical companies to mitigate the risks associated with drug development typically include product portfolio diversification, adaptive trial design, and collaboration with academic institutions. Academic institutions often face challenges in target validation and subsequent development, hindered by complex funding application processes and limitations in technology platforms (Dorsch et al., 2015). Although large pharmaceutical companies possess the necessary expertise, technology, and resources for new drug development, innovative targets frequently originate from more specialized entities like universities, research centers, or biotech firms (Melese et al., 2009). Therefore, collaboration between these diverse institutions can enhance the efficiency of new drug development and lower R&D costs. As biotechnological advancements continue, the need for collaboration between academia and industry grows. Hence, intensified interaction and partnerships between these sectors can bridge the gap between basic research and drug development, swiftly converting new targets into marketable products, which is crucial for the future success of new drug R&D (Yu, 2016; Rosenblatt, 2013).

The shift from the era of chemical drugs to biologics has transformed the academic research paradigm in new drug discovery, driven by advancements in big data and biotechnology. For instance, drug screening predominantly identified drug candidates during the chemical drug era, whereas target-based drug design has emerged as a key approach for small molecule drug discovery in the biologics era, supported by advances in molecular and structural biology (van Montfort and Workman, 2017; Campos et al., 2019). The advent of next-generation sequencing (NGS) and CRISPR technologies has also brought about a revolutionary change in the field of drug development. NGS technologies have enhanced the success of clinical trials by analyzing the genomic information of individuals and identifying patient populations that are more likely to respond to specific therapeutic regimens (Goodwin et al., 2016). CRISPR technologies have facilitated the creation of accurate disease models, accelerating the validation of drug targets and enabling the development of more precise and personalized treatments (Pacesa et al., 2024). The integration of new technologies like big data platforms and analytics has improved the sharing of digital resources, accelerating the completion of clinical trials by facilitating access to clinical trial data and real-world research data, thus speeding up the approval of new drugs (Evangelatos et al., 2016; Miller et al., 2019). Furthermore, artificial intelligence (AI) techniques are extensively employed in the predictive analysis of drug design and modeling of drug interactions (Vo et al., 2022).

The development of the Internet and transportation networks has also greatly facilitated international collaboration. For example, global multicenter clinical trials not only shorten the time to market for new drugs but also increase drug accessibility for patients, minimize redundant clinical trials, and reduce the waste of R&D resources (Finfer et al., 2021). Additionally, the cross-border mobility of researchers brings new perspectives to research organizations, mitigates academic inbreeding, broadens research networks, and boosts scientific impact (Aykac, 2021). These advancements in digitalization and information technology have profoundly enhanced the development of scientific research carriers towards networking, platformization, and connectivity. This evolution in research cooperation has enabled networked collaborations and is likely to lead to an integrated scientific research model that spans from basic research to technology development (Yang et al., 2021; Wang et al., 2022).

The emergence of breakthrough technologies and the increasing demand to address unresolved diseases have motivated researchers to explore the evolution of academic and industrial collaborations in new drug R&D. Thus, using the development of two lipid-lowering drugs as case studies, we first classified the publications within the drug R&D academic chain by different types of knowledge innovation. We then examined the evolutionary trends in scientific research cooperation at the levels of authors, institutions, and countries. Additionally, we also analyzed existing barriers to collaboration and offer recommendations for enhancing cooperative efforts in drug development.

## 2 Objects and methods

### 2.1 Research objects

**FIGURE 1:**
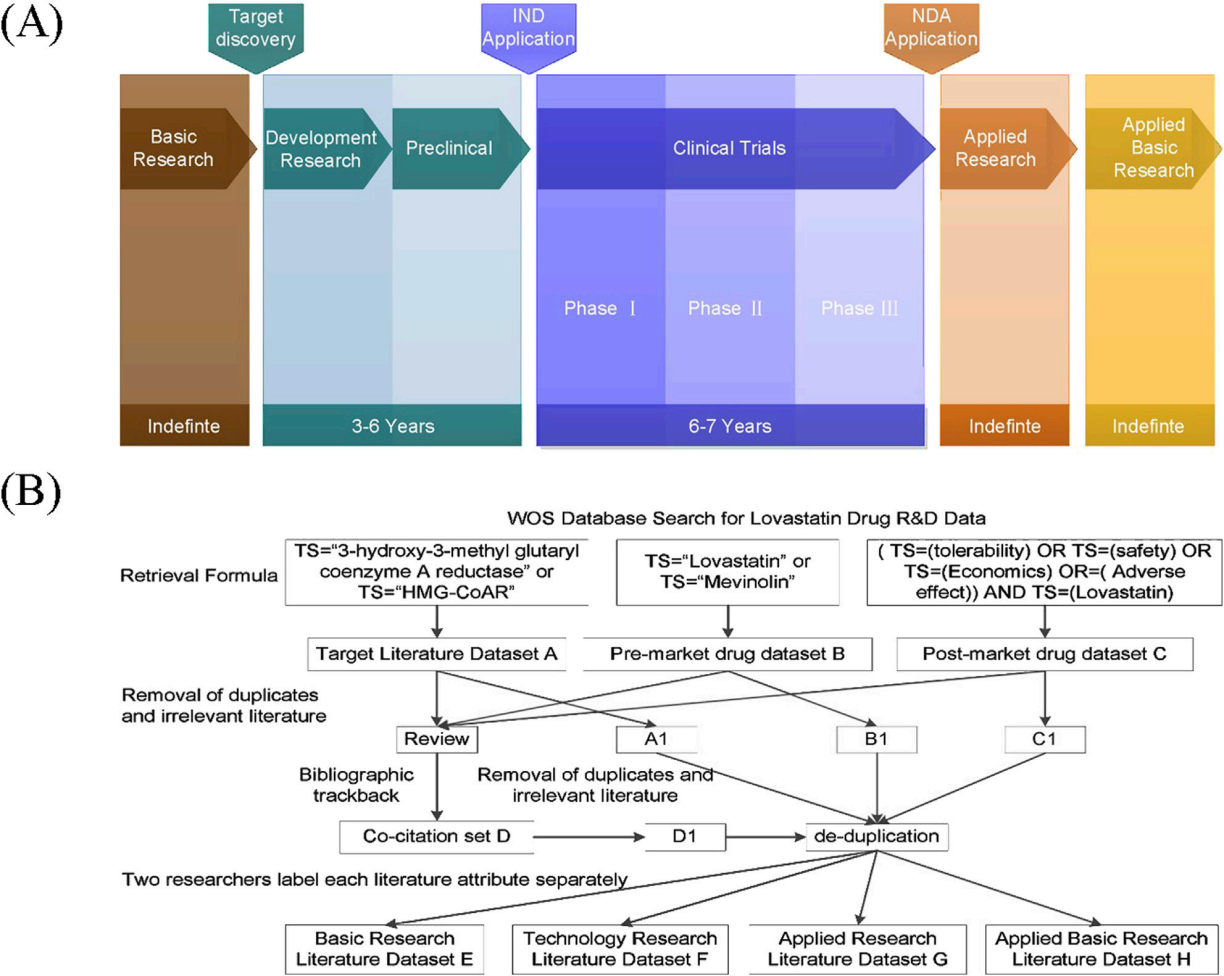
Integrated framework of new drug research and development with data processing workflow. **(A)** schematic of the full-cycle drug discovery pipeline; **(B)** flowchart of data retrieval and analysis.

The development and marketing of new pharmaceuticals must undergo a thorough evaluation process, including the discovery of new chemical entities or novel action targets, followed by preclinical and clinical research, and eventually regulatory approval and post-marketing surveillance (FDA, 2021). The entire chain of new drug R&D can be segmented into six stages: Basic Research, Development Research, Preclinical Research, Clinical Research, Applied Research, and Applied Basic Research. The initial five stages are inevitable, whereas the ultimate stage may or may not occur subsequent to the drug’s market release (Figure 1A). The end products are the drugs that reach the market, yet the knowledge innovation during their development is documented through published research papers, filed patents, and the submission of clinical research and new drug applications. This study focuses on research papers and patents as indicators of research collaborations in new drug R&D.

Cardiovascular disease is the leading cause of death worldwide and is also the primary cause of mortality from major diseases in China (World health statistics, 2024). Dyslipidemia, a key risk factor for cardiovascular disease, necessitates early pharmacological intervention to halt disease progression. Introduced in the 1980s, HMG-CoA reductase inhibitors (statins) marked the first generation of lipid-lowering medications. Despite being on the market for over 3 decades, statins continue to be one of the most frequently used medications in clinical settings. The advent of biotechnology, particularly advances in synthetic biology at the start of the 21st century, opened new avenues for developing lipid-lowering drugs. Evolocumab, which was marketed in 2015, is a representative of the new generation of lipid-lowering drugs, targeting the new target PCSK9, which is one of the auxiliary drugs in clinical application. Both drugs are the first lipid-lowering drugs developed and marketed for new targets, a typical example of the era of chemical drugs and biologics. Therefore, the evolution of research collaboration in new drug R&D can be analyzed by comparing the current status of research collaboration for these two drugs.

### 2.2 Research methods

The classification framework for the entire academic chain of new drug R&D was established through expert interviews and group discussions. Initially, experts specializing in lipid-lowering drug research from various fields such as basic medicine, drug development, clinical medicine, epidemiology, and medical research management were chosen for interviews. Subsequently, the researchers compiled and organized the information from these interviews into a preliminary draft. This was followed by a discussion among the expert group, during which the initial draft was revised and refined to produce the final version.

Social network analysis was employed to examine the collaborative relationships in drug R&D across countries/regions, institutions, and authors. Collaborations were categorized into nine types based on the author’s country/region and their affiliated organization. These categories include solo authorship (the paper has only one author listed), inter-institutional collaboration (the authors of the paper are affiliated with different institutions), multinational or regional collaboration (the authors of the paper are located in different countries or regions), university collaboration (all the collaborating institutions in the paper are universities), enterprise collaboration (all the collaborating institutions in the paper are enterprises), hospital collaboration (all the collaborating institutions in the paper are hospitals), collaborations between universities and enterprises (the collaborating institutions in the paper include universities and enterprises), collaborations between universities and hospitals (the collaborating institutions in the paper include universities and hospitals), and tripartite collaborations involving universities, enterprises, and hospitals (the collaborating institutions in the paper include universities, enterprises, and hospitals).

### 2.3 Data retrieval

The research on lovastatin and evolocumab was conducted using the Web of Science database, as depicted in the data retrieval and processing flowchart shown in Figure 1B, using lovastatin as an example. Initially, literature related to the target (Set A) and those pertaining to drug development and application (Sets B and C) for each drug were collected. To ensure a comprehensive review of relevant academic outputs, references from the retrieved review articles were traced back to create a co-citation dataset (Set D). After eliminating duplicates and irrelevant entries, two researchers independently reviewed the titles and abstracts to categorize the type of research. Discrepancies or uncertainties in labeling were resolved through discussion with a third researcher. Ultimately, the literature was organized into Basic Research (Set E), Technology Research (including drug development, preclinical, and clinical studies) (Set F), Applied Research (Set G), and Applied Basic Research (Set H). Retrieved 30 December 2023.

The evolocumab search formula used was as follows: dataset A: TS = “proprotein convertase subtilisin kexin9” or TS = “PCSK9”, time: 2003–01–01 to 2015–08–27; Dataset B: TS = “evolocumab” or TS = “AMG145” and (Article or review), from 2003 to 01 -01 to 2015–08–27; Dataset C (((TS=(tolerability)) OR TS = (safety)) OR TS = (Economics) OR = (Adverse effect)) AND (TS = (evolocumab) OR TS=(repatha)), time: 2015–08–27 to 2023–12–31.

When variations in capitalization, abbreviations, or full names occur in author names, organization names, and country names, these were manually adjusted to conform to a standardized format. Authors sharing the same name were treated as distinct individuals if they are affiliated with different institutions. All data used in this study were derived from the publicly accessible academic database Web of Science, with its collection and distribution adhering to relevant ethical guidelines. There was no risk of re-identification of personal or sensitive information.

## 3 Categorization each paper into different links of the academic chain

The outcomes of knowledge innovation in new drug R&D include identifying and validating drug targets; discovering and refining initial and lead compounds; selecting and formulating drug candidates; gathering pharmacokinetic, pharmacodynamic, toxicological, and pharmacological data from animal and clinical trials; evaluating tolerability, safety, and adverse reactions; and analyzing cost-effectiveness. The experts agreed that papers can be classified based on the research purpose, research object, research method, research result, research significance, subject field and data source of each paper. The classification framework is depicted in Table 1. In practice, researchers involved in classification should possess a pharmaceutical background and undergo specialized training before undertaking this task.

**TABLE 1:** The attribute division basis of a single paper in the field of new drug research and development.

Classification entry	Basic research	Drug development research	Preclinical research	Clinical research	Applied research	Applied basic research
Research Purpose	Discover underlying principles	Screen or design molecular structures with therapeutic potential	Evaluate the efficacy and safety of drug candidates in animal models	Assess the efficacy and safety of drug candidates in human populations	Determine the real world efficacy and safety of new drugs	Identify previously unrecognized mechanisms of safety risks or efficacy in newly launched drugs
Research Subjects	Experimental animals, cells, populations	Compound databases, antibody libraries, mutant libraries, siRNA databases, *etc.*	Animal models for preliminary testing	Healthy volunteers and target patients for candidate drug therapy evaluations	Target patients for assessing new drug therapy	Experimental animals, cells
Research Methods	Animal and, cell experiments, epidemiological studies, *etc.*	High-throughput screening, structure- or ligand-based drug design, structure-activity relationship analysis, formulation development, *etc.*	Studies on pharmacodynamic, pharmacokinetics, toxicology, immunogenicity, *etc.*	Phase I-III clinical trials, bioequivalence trials, *etc.*	Phase IV clinical trials, randomized controlled clinical trials, questionnaire studies, real-world studies, *etc.*	Further animal and cell experiments
Research Goals	Discover and determine causes and targets of diseases	Identify potential drug candidates	Elucidate the relationships between plasma concentration and time, *in vivo* ADME processes, dose-effect, time-effect, administration routes, dosage forms, dosing regimens, efficacy evaluation, and side effects	Clarify the pharmacokinetics, safety, efficacy, optimal dosage and dose range, and side effects of drug candidates in humans	Determine the safety, effectiveness, cost-effectiveness, drug interactions, and potential new uses of new drugs	Investigate the mechanisms of adverse effects and explore additional indications for new drugs
Research significance	Uncover disease patterns and identify therapeutic targets	Screen or design drug candidates and address key technological aspects of drug synthesis	Provide a reliable basis for clinical research	Ensure that new drugs are safe and effective for clinical use, providing scientific evidence for their application	Enhance better understanding of the properties of new drugs and ensure their safety, efficacy and cost-effectiveness	Ensure the safety of drugs and provide scientific evidence for expanding the application range of drugs
Discipline Fields	Molecular Biology, Endocrinology and Metabolism, Cell Biology, Pharmacology and Pharmaceutical Sciences, Physiology, Cardiovascular System and Cardiology, Genetics and Heredity, Developmental Biology, *etc.*	Medicinal chemistry, organic chemistry, biochemistry, computational chemistry, analytical chemistry, structural biology, bioinformatics, synthetic biology, *etc.*	Pharmacology and pharmacology, drug analysis, pharmaceutical preparation, toxicology, pharmacokinetics, immunology, genetics, *etc.*	Clinical pharmacology, clinical pharmacokinetics, clinical medicine, medical imaging, laboratory medicine, nursing, medical statistics, *etc.*	Clinical medicine, social medicine, rehabilitation medicine, pharmaceutical economics, epidemiology, medical ethics, medical statistics, *etc.*	Physiology, cell biology, toxicology, pharmacology and pharmacy, pharmacokinetics, *etc.*
Data sources	Experimental studies, epidemiologic research	Databases, experimental studies	Experimental studies	Clinical trials	Clinical trials, research data, public data	Experimental studies

## 4 Analysis of thesis collaboration in new drug R&D

### 4.1 Changes in collaboration rate

**FIGURE 2:**
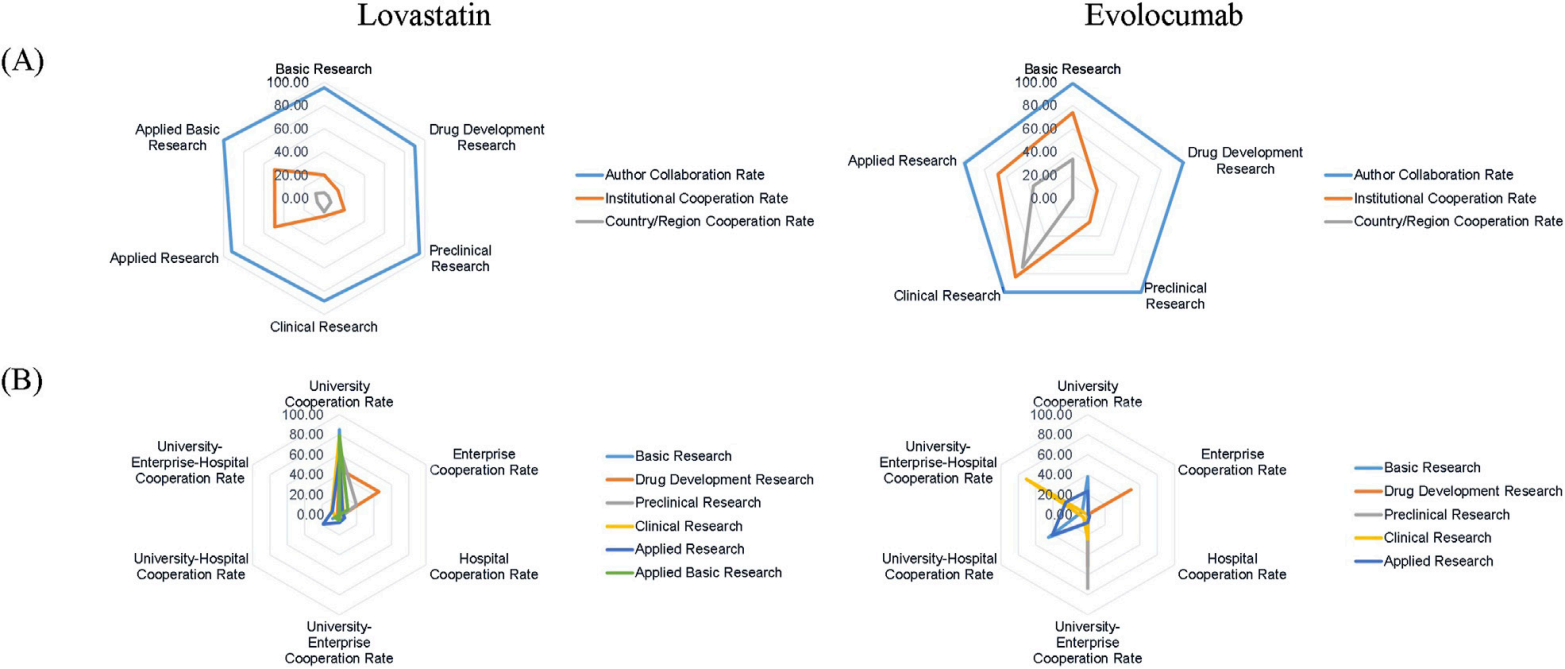
Distribution of collaboration patterns in published research. **(A)** Collaboration rates at author, institutional, and national/regional levels; **(B)** Collaboration frequency across institution types.

The collaboration rates among research papers for both drugs were ranked as follows: authors, institutions, then inter-country/inter-regional collaborations. However, evolocumab exhibited a substantial rise in both institutional and inter-country/territorial collaborations compared to lovastatin, with the most significant increase observed in clinical studies and the least in preclinical and drug development studies (Figure 2A). The primary collaborating institutions vary across different stages of the academic chain. University collaboration played a crucial role in the R&D of lovastatin. In contrast, the types of institutional cooperation in the R&D of evolocumab varied by stage. For instance, university-enterprise-hospital collaborations were most prevalent in clinical studies, while enterprise collaborations dominated in drug development studies (Figure 2B). This trend indicates that sole university collaboration is no longer the predominant model in new drug R&D, giving way to a rise in diverse inter-institutional collaborations.

### 4.2 Comparison of the sizes of cooperation

**FIGURE 3:**
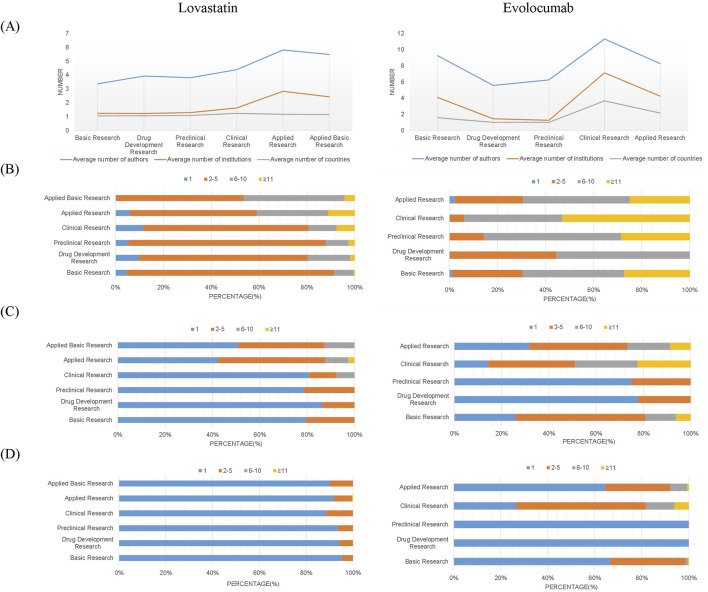
Distribution of collaboration scale across authors, institutions, and countries/regions. **(A)** Average number of authors, institutions, and countries/regions per publication; **(B)** Author-level collaboration scale; **(C)** Institutional collaboration scale; **(D)** Cross-national/regional collaboration scale.

The average number of authors and institutions involved in drug R&D papers exhibited consistent trends across different stages of the academic chain. Specifically, lovastatin research saw the largest collaboration size in applied research, while evolocumab’s largest collaborations occurred in clinical research, with evolocumab generally having larger collaboration sizes than lovastatin (Figure 3A). An in-depth analysis of the collaboration size reveals trends in research team sizes throughout the evolution of the academic chain. Based on the size of the collaborations, teams can be classified as independent, small, medium, or large. In terms of author collaborations, small teams of 2-5 members predominated in the full academic chain for lovastatin studies, whereas medium-sized teams of 6–10 members were more common in evolocumab research (Figure 3B). Regarding institutional collaborations, both drugs typically saw intra-institutional collaborations dominate in drug development and preclinical stages, while applied research often involved collaborations between 2-5 institutions. However, for evolocumab, both basic and clinical research stages also predominantly featured collaborations of 2-5 institutions, contrasting with lovastatin, which mainly had intra-institutional collaborations (Figure 3C). In terms of country/region collaborations, over 80% of lovastatin studies were conducted within a single country/region, whereas evolocumab clinical studies frequently involved 2-5 countries/regions (Figure 3D). This pattern indicates that as scientific research becomes more specialized, mid-sized and large teams are increasingly necessary across all stages of new drug development. Additionally, clinical and applied studies often require collaborative efforts across multiple organizations and countries.

### 4.3 Citation impact analysis of collaboration

**FIGURE 4:**
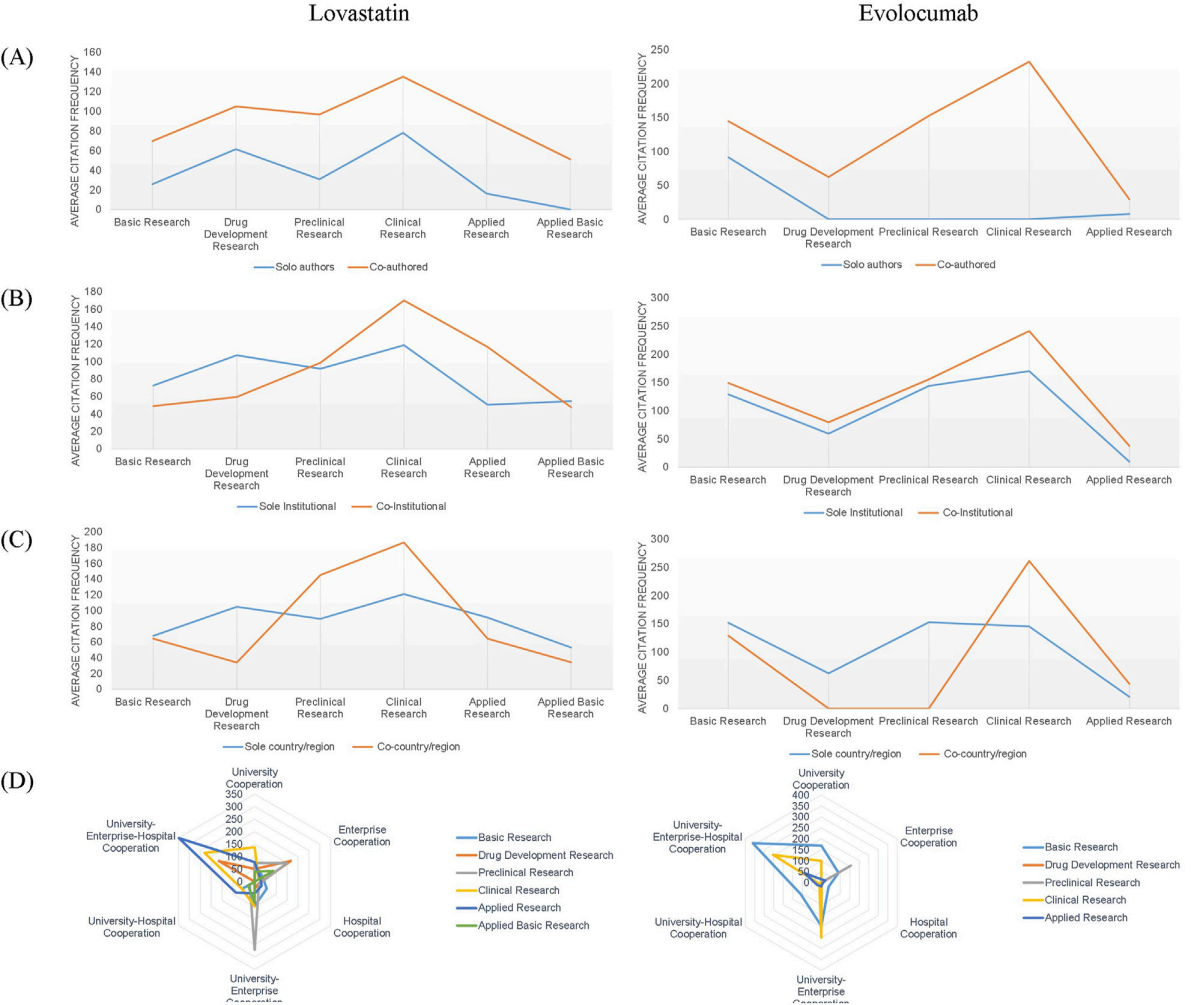
Variations in average citation frequency across authors, institutions, countries/regions, and institution types. **(A)** Author-level citation trends; **(B)** Institutional-level collaboration impact; **(C)** National/regional citation dynamics; **(D)** Citation patterns across institution types.

We calculated the average citation frequency of collaborative papers compared to papers authored by a single researcher to evaluate the influence of collaboration type on the potential impact of a paper, across various research types of authors, institutions, and countries/regions. According to Figure 4A, the average citation frequency of papers with multiple authors was consistently higher than that of papers authored by a single individual across all stages of the academic chain for both drugs, with the highest frequencies observed in clinical studies. For lovastatin, papers resulting from multi-institutional collaborations had higher citation frequencies than those from single institutions, particularly in clinical and applied research. In the case of evolocumab, multi-institutional papers outperformed single-institution papers in terms of citation frequency across all stages of the academic chain (Figure 4B). Papers involving multi-national or multi-regional collaborations also garnered higher citation frequencies compared to those involving single countries or regions, particularly in clinical research for both drugs (Figure 4C). As shown in Figure 4D, papers resulting from collaborations between universities, enterprises, and hospitals, as well as those from enterprise-alone and university-enterprise collaborations, were associated with higher impact research outputs.

### 4.4 Comparison of national/regional collaboration networks

**FIGURE 5:**
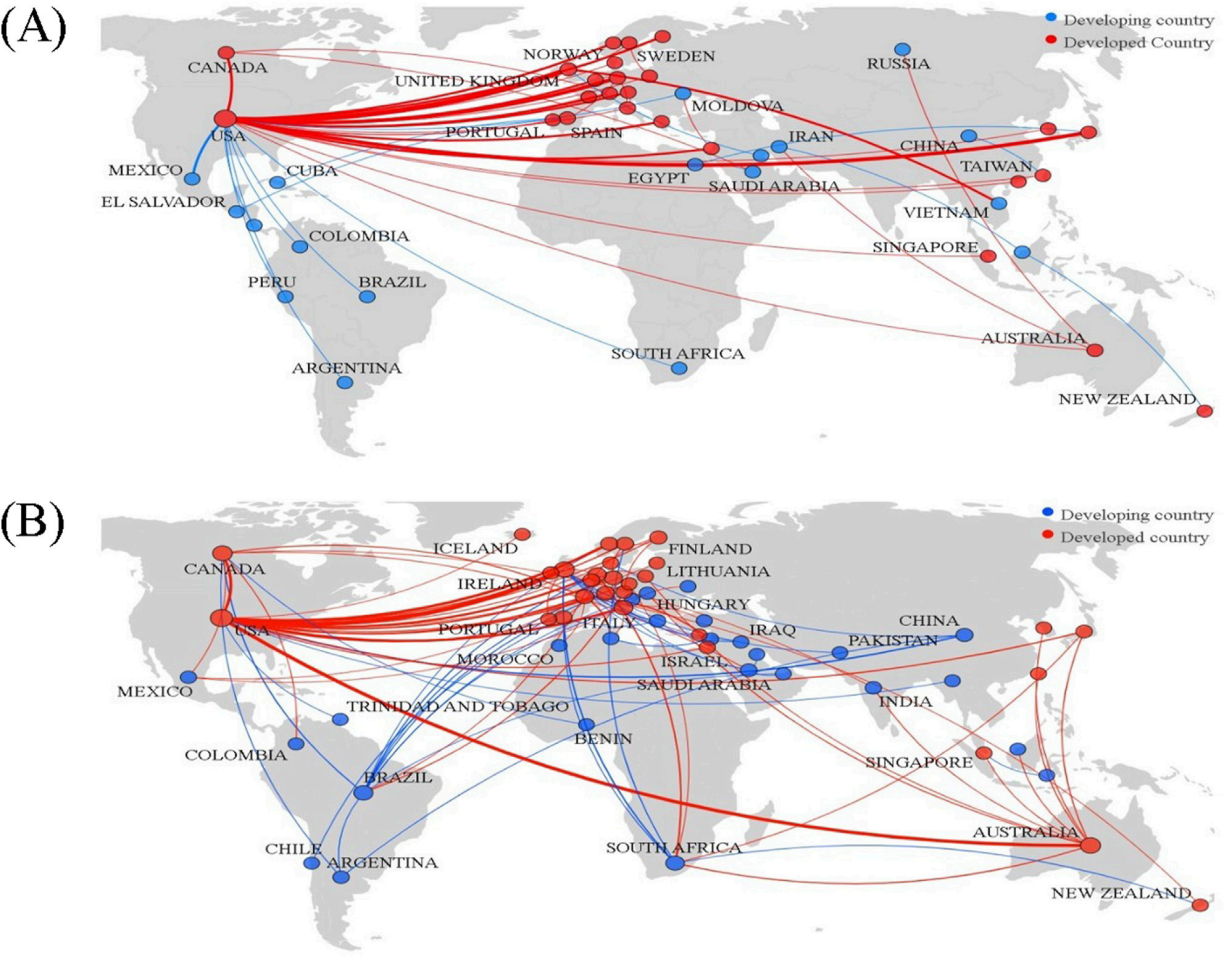
Visualization of national/regional collaborative networks. **(A)** Lovastatin; **(B)** Evolocumab.

The collaboration network for evolocumab research expanded to include 55 countries, marking an increase of 12 new countries compared to lovastatin research. In both drug studies, the U.S. and the U.K. consistently ranked among the top five for both degree centrality and betweenness centrality, highlighting their central role in lipid-lowering drug research. As depicted in Figure 5, developed nations like the US, UK, Germany, Canada, Australia, Italy, the Netherlands, and Norway are at the center of the collaboration network, with each maintaining strong cooperative ties with the United States. Conversely, developing countries such as China, South Africa, Mexico, and Brazil are positioned on the periphery of this network. A further breakdown of country collaborations along the academic chain showed reduced collaboration in drug development and preclinical research stages. For lovastatin, collaboration primarily occurred among countries like the US, Japan, and Canada, whereas for evolocumab, it is mostly between the US and the UK. Applied research featured the most extensive country collaborations and the closest partnerships, followed by basic research. The US remained a central figure across all stages, while countries like the UK and Australia are more prominent in basic and applied research, and developing countries such as South Africa were more active in clinical research.

### 4.5 Comparison of institutional collaboration networks

Table 2 compares the key metrics of the institutional collaboration networks for the development of lovastatin and evolocumab. The network for evolocumab included more nodes and edges compared to lovastatin, suggesting a larger and more complex network. This increased network density for evolocumab indicates a higher number of connections between nodes, leading to more frequent and intense information flow. Despite network of evolocumab having a larger diameter, it featured shorter average path lengths, which typically means quicker information transfer across the network. Additionally, a higher clustering coefficient in network of evolocumab suggested that institutions tend to form closely-knit groups or clusters. Collectively, these characteristics demonstrate that evolocumab’s institutional collaboration network has a more complex structure and was overall more efficient, with faster information dissemination and better robustness to complex networks.

**TABLE 2:** Overall network characteristics of institutional cooperation networks.

Drug name	Number of nodes	Number of edges	Network density	Maximum number of subnet nodes (%)	Network diameter	Average path length	Clustering coefficient
Lovastatin	535	920	0.00322	284 (53.08)	9	4.341	0.239
Evolocumab	770	2,580	0.00436	653 (84.80)	12	4.127	0.258

**FIGURE 6:**
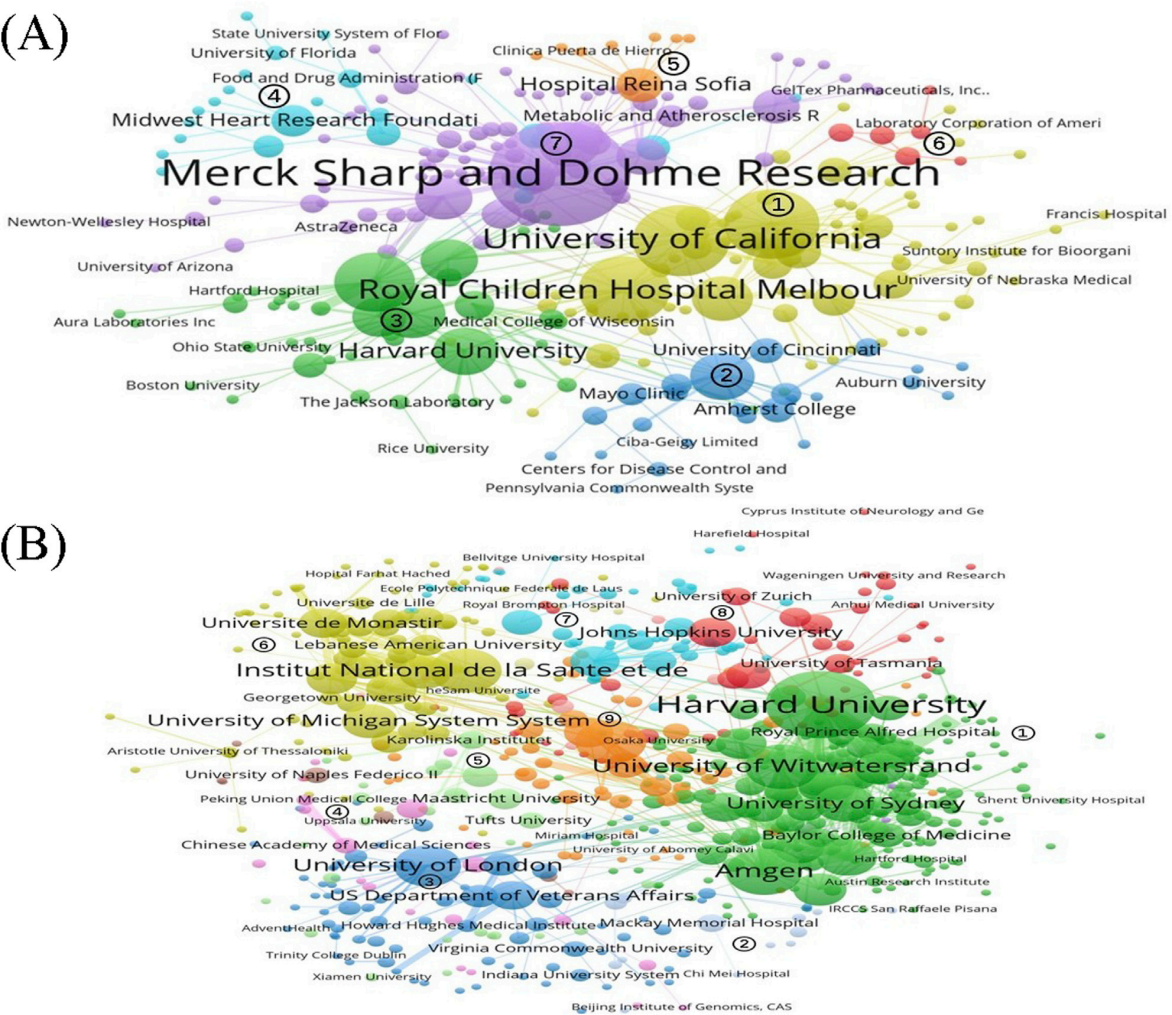
isualization of institutional collaborative networks **(A)** Lovastatin; **(B)** Evolocumab.

Figure 6 illustrates the institutional collaboration network for the two drugs, where the node size reflects the number of publications issued by each institution, and the line thickness indicates the frequency of collaboration between institutions. The Louvain algorithm was used for clustering, with different node colors representing the various institutional clusters. For lovastatin, institutions in clusters 1, 2, and 3 are predominantly universities or research institutes, such as the University of California, the University of Texas, and the Royal Children’s Hospital in Melbourne, which primarily engage in basic and applied research. Clusters 4, 5, and 6 comprise a mix of universities, foundations, hospitals, and companies, focusing on applied and applied basic research. Specifically, Cluster 2 is anchored by Merck Sharp & Dohme and includes a diverse group of institutions like hospitals, universities, and research institutes, which are involved in a broad range of activities including developmental, preclinical, clinical, and applied research (Figure 6A). The institutional collaboration network for evolocumab is divided into nine distinct clusters. Cluster 1, the largest of these groups, comprises a diverse array of institutions including universities, companies, hospitals, and foundations from countries like the United States, South Africa, Australia, and the United Kingdom. This cluster primarily engages in a comprehensive range of research activities encompassing basic, developmental, preclinical, clinical, and applied research. Clusters 2 and 3 are predominantly made up of universities, research institutes, and hospitals focused on basic and applied research. The main institutions in clusters 4 and 5 consist of universities located in China, Germany, the Netherlands, and other countries, also concentrating on basic and applied research. Clusters 6 and nine include a mix of universities, research institutes, and hospitals from countries such as France and Canada, mainly involved in basic research, clinical research, and applied research. Finally, clusters 7 and 8 feature hospitals and schools from countries like Spain, Switzerland, and Australia, focusing on basic and applied research activities (Figure 6B).

### 4.6 Comparison of author collaboration networks

The overall parameters of the author collaborative network in both drug developments are shown in Table 3. Evolocumab R&D paper’s author collaboration network has more nodes and edges, which represents a larger network with more authors and connections. The smaller network density indicates that nodes in this network are not connected to all other nodes. Furthermore, the network’s smaller diameter and shorter average path lengths, compared to those of the lovastatin R&D paper’s author network, imply that the distance between any two nodes is shorter, leading to tighter connections and quicker dissemination of information or resources across the network. These attributes indicate that the author collaboration network for evolocumab is structurally more complex and efficient, facilitating faster information flow.

**TABLE 3:** Overall network characteristics of author collaboration networks.

Drug name	Number of nodes	Number of edges	Network density	Maximum number of subnet nodes (%)	Network diameter	Average path length
Lovastatin	2,498	4,608	0.00076	495 (20.1)	21	8.666
Evolocumab	3,341	8,022	0.00071	1896 (56.7)	20	7.391

The participation of authors throughout the academic chain was analyzed by tracking the percentage of authors who remained active across different stages of the chain. For lovastatin, 9.17% of authors engaged in basic research continued contributing to later stages, with 6.2% focusing specifically on preclinical research. Among those involved in preclinical studies, 8.5% also contributed to development research, while 18.4% of authors in clinical research were active in applied research as well. In the case of evolocumab, 5.6% of authors from basic research remained active in subsequent stages, with 4.6% concentrating on applied research. Remarkably, 36.6% of authors in development research had previously been involved in preclinical research, and 54.7% of those in clinical research continued to publish in applied research. This analysis indicates a significant overlap of authorship between development and preclinical research, as well as between clinical and applied research, highlighting increased continuity among authors across these stages.

## 5 Discussion

### 5.1 Scientific collaboration can lead to more citations, especially in clinical research

Prior research has demonstrated that collaborative efforts result in enhanced paper impact, primarily due to an increase in visibility, broader dissemination, and the implementation of more expansive and comprehensive research designs (Dusdal and Powell, 2021; Dong et al., 2018). The citation impact analysis of collaborations demonstrates that papers authored collaboratively by different authors, institutions, and countries/regions achieve higher average citations in clinical studies. This trend is largely due to the pivotal role of clinical research in validating the safety and effectiveness of new pharmaceuticals, garnering significant interest from both the academic and industrial sectors. Additionally, clinical studies often introduce novel treatments, drugs, or therapeutic approaches that require multi-party collaboration, leading to more innovative outcomes and enhanced academic value (Liang et al., 2019).

In the realm of basic research, collaborative efforts yield higher average citations than in applied research. This increase could be attributed to basic research laying the scientific groundwork for new drug development, advancing our understanding and developing new methods to combat diseases through groundbreaking scientific findings and innovative technological advancements, with a strong interdisciplinary approach (Valentin et al., 2016). Papers resulting from collaborations between universities, businesses, and hospitals exhibit higher citation rates compared to other types of institutional collaborations. It is conceivable that commercial papers have greater potential for conversion into tangible assets, as evidenced by prior research in the field, with business-related papers achieving greater prominence and receiving denser citation from patents than academic papers (McManus et al., 2021).

### 5.2 Developing countries have increasingly participated in the R&D of new drugs

The growing involvement of various countries and regions in the international collaboration for new drug research and development is linked to the global aging population. As the elderly demographic expands, there is an increased need for new therapeutic methods due to evolving disease patterns, leading to a significant demand for innovative drugs. In addition, this surge in collaboration is also supported by global governmental policies promoting drug development and the widespread nature of clinical trials (Thiers et al., 2008; Seifirad and Alquran, 2021). This is mainly due to the fact that developed countries possess sophisticated scientific and technological infrastructures along with robust R&D capabilities, supported by government and corporate policies, including financial backing. On the other hand, developing countries and regions, such as China, South Africa, and Brazil, though initially on the fringes of these cooperative networks, have shown marked increase in their participation. According to the IQVIA Institute’s Global Trends in R&D 2024 report, China has emerged as a key contributor to global pharmaceutical innovation, which can be attributed to the worldwide trend towards scientific collaborations and the strengthening of research capacities in these nations (Braga, 2021). Additionally, there is a current issue with a lack of diversity in national/regional partners throughout the academic stages of R&D for certain drugs. This is primarily attributable to the fact that international R&D collaboration is currently confronted with a number of specific challenges, including regulatory barriers, issues pertaining to intellectual property rights and logistical challenges associated with the conduct of multinational or multicultural clinical trials. Moving forward, it is crucial to develop new channels for multilateral research collaborations among different countries and regions. Developing countries can actively set up special funds to support international cooperation. They can also establish well-defined channels for transferring knowledge from developed countries to developing ones. For example, joint laboratories and data-sharing platforms can help make up for the lack of resources in basic research in developing countries, enabling them to grasp the opportunities in new drug development.

### 5.3 Diversification of institutional cooperation modes helps resource sharing and complementary advantages

We are currently living in an important era of biologics R&D. Institutional collaborations show varying degrees of engagement across different stages of the academic chain. For instance, 23.61% of the papers in development and preclinical studies involve collaborations between two or more institutions, whereas this figure rises to 74.99% in other types of academic studies. This discrepancy may stem from the commercially sensitive nature and intellectual property concerns associated with new drug development, which often require a high level of technological confidentiality (Courage and Calzavara, 2015). As a result, the necessity for institutional collaboration differs across various segments of the academic chain, with cross-institutional collaborations in basic, clinical, and applied research emerging as the predominant form. Further analysis of institutional collaboration types indicates a shift away from solely university-centric collaborations towards more diverse partnerships involving universities, enterprises, and hospitals. Previous studies have found that collaborative networks in drug development are dominated by pharmaceutical companies (Cheng et al., 2020). Such collaborative models facilitate rapid information exchange, resource sharing, and the pooling of complementary strengths between the academic and industrial sectors, thereby enhancing the overall progression of the pharmaceutical and healthcare industries (Liu et al., 2024). This trend is also linked to recent changes in the business strategies of major pharmaceutical firms, which are increasingly sourcing innovation from academic institutions alongside their internal research efforts (Rosenblatt, 2013). Furthermore, there are challenges in the collaboration between academia and industry, including the management of intellectual property and the existence of cultural differences between these two environments.

### 5.4 Institutional collaboration shows strong similarity and proximity effects

In our analysis of cluster patterns within collaborative networks, we noted an intriguing trend: research institutions within the same cluster often concentrate on similar segments of the academic chain and are typically located near one another. This observation highlights two significant dynamics in institutional collaboration: the similarity effect and the proximity effect. The similarity effect is manifested in the consistency of the collaborating institutions in terms of knowledge domains, technical expertise, and research interests. This facilitates communication and collaboration among them and promotes the achievement of common research goals, similar to the findings of previous studies (Anckaert and Peeters, 2023). Conversely, the proximity effect suggests that being geographically closer lowers collaboration costs and boosts the likelihood of regular in-person interactions, potentially leading to more productive collaborations (Ostergaard and Drejer, 2022). The combination of geographic closeness and shared academic interests and disciplines further solidifies these inter-institutional relationships, fostering enhanced knowledge exchange and innovation.

### 5.5 Reduced author collaboration between basic research and subsequent stages of the academic chain

In the current era of “big science,” the importance of resource sharing and collaborative research has grown significantly. The widespread use of the Internet has reduced the costs associated with cross-regional collaborations, enabling the formation of large-scale teams (Hu et al., 2020), and facilitating broader coverage across various segments of the academic chain. There has been an increase in the proportion of the same authors involved across the development, preclinical research, clinical, and applied research segments. This trend supports the rapid translation of discoveries from the laboratory to clinical settings and bridges the gap between scientific innovation and industrial R&D, thereby improving the overall efficiency of the academic chain. However, there is a notable lack of collaboration between authors in basic research and those in later stages of the academic chain. This separation can lead to issues such as information asymmetry, misunderstanding, and barriers in the application process, ultimately impacting the efficiency and speed of translating scientific discoveries into technological achievements, echoing findings from previous studies on the inefficiencies of university technology transfers (Ma et al., 2022). Despite the foundational role of basic research in fostering major innovations and applied research, there remains a disconnect with applied research and technological development. This highlights the need for establishing effective communication mechanisms to bridge the gap between basic research and applied development.

A number of factors currently impede collaboration between the basic research and applied phases of new drug R&D. These include publication pressures, cultural differences between disciplines and barriers to technology transfer. The pressure to publish may prompt researchers to prioritise short-term results over longer-term, potentially higher-impact basic research (Lv, 2018). Moreover, collaboration between basic and applied research necessitates the involvement of experts from disparate disciplinary fields. However, the existence of cultural and methodological divergences between these disciplines may impede effective communication and collaboration (Kluger and Bartzke, 2020). And issues may arise pertaining to the absence of efficacious technology transfer mechanisms and the dearth of expertise in the technology transfer process. In addition, discrepancies between the scientific inquiry and commercial objectives of the respective parties may also constitute an obstacle to collaboration. It is of the utmost importance to promote a more integrated academic chain from a policy perspective in order to accelerate the innovation cycle and facilitate the development of new therapies in the pharmaceutical industry.

## 6 Conclusions and recommendations

This study concluded that the new drug R&D research collaboration patterns evolve spontaneously with productivity updates. In the future, it is necessary to enhance the involvement of pharmaceutical companies in the essential research phase of new drug developments, continuously strengthening the relationships across all segments of the academic chain and thoroughly boosting the efficiency of transforming new drug R&D into practical applications.

As the costs and risks associated with technology development increase, research entities such as universities, research institutes, hospitals, pharmaceutical companies, and emerging biotech firms have increasingly begun to collaborate more actively. By leveraging their unique strengths, these organizations aim to pool resources and specialize their roles to expedite the new drug development process. The following recommendations are put forth for consideration: 1. The development of quantitative indicators to measure the extent and level of innovation elements present throughout the entirety of the academic chain associated with the R&D of new drugs is advised. 2. The improvement of quantitative indicators to measure the efficacy and efficiency of new drug R&D collaborative networks is recommended in order to provide more precise benchmarks for success. 3. With an increase in productivity, it is advised that the mode of research collaboration in new drug R&D be proactively adjusted in order to align more closely with market demands.

Large pharmaceutical firms often have access to extensive industry data, offering rich, empirical insights for academic studies. Additionally, ample R&D funding from these companies can support more expansive research efforts. Companies with robust independent R&D capabilities often hold advanced technological expertise as well. Deepening collaboration among universities, hospitals, and pharmaceutical companies can foster technical exchanges, boost research innovation, push the boundaries of scientific discovery, and ultimately improve the quality and impact of research. Specific suggestions for cooperation include the following: 1. Pharmaceutical companies should establish target validation grant funds to quickly identify innovative targets with developmental potential; 2. Establishment of joint research institutes between universities, hospitals and enterprises based on strategic partnerships; 3. Creation of an information-sharing platform for the discovery and development of new drugs based on advanced technologies such as big data and AI.

Technology research and applied basic research serve as critical conduits for the transition between basic and applied research, playing a pivotal role in advancing key core technologies. These efforts not only enable the efficient conversion of research findings into tangible outputs but also supply foundational knowledge for addressing specific challenges. Thus, bolstering collaboration among authors, institutions, and nations across various research domains is crucial for optimizing the academic chain’s efficiency. Specific recommendations are as follows: 1. Proactively identify key researchers engaged in basic, technology, or applied research. Focus on their development into leaders in technological innovation management, supporting their crucial role in transitioning from drug target discovery to clinical application; 2. Establish a cross-regional and cross-link “university-enterprise-hospital” integration platform, with a strong leadership role for enterprises to deepen inter-institutional collaboration; 3. Overcome political and economic challenges in international cooperation to strengthen multilateral collaboration among countries and regions within the academic chain.

## 7 Limitations

This study selected the development of two types of lipid-lowering drugs as a case study to examine the impact of innovation on the evolutionary trends of collaborative networks. Due to the distinct research foundations and historical development of different drug classes, this analysis might encounter some degree of extrapolation error. The field of oncology drugs, in particular, which evolves rapidly, might demonstrate more intense and complex patterns of research collaboration. In the future, the scope of this analysis will be broadened to include multiple drug types, aiming to mitigate this limitation and enhance the understanding of collaborative evolution in drug R&D. This expansion will facilitate a systematic examination of the similarities and differences in collaboration evolution across various drug classes, offering a more detailed view of the dynamic evolution of collaboration networks in drug R&D. In addition, the reliance on available publication and patent data could limit insights, as not all research activities or collaborations are publicly documented.

## Data Availability

The original contributions presented in the study are included in the article/supplementary material, further inquiries can be directed to the corresponding author.
